# Lysine lactylation (Kla) might be a novel therapeutic target for breast cancer

**DOI:** 10.1186/s12920-023-01726-1

**Published:** 2023-11-10

**Authors:** Jian Deng, Xinyi Liao

**Affiliations:** 1 Department of Thyroid Breast Surgery, The Second Affiliated Hospital, Hengyang Medical School, University of South China, No. 35 Jiefang Avenue. Hengyang, Hengyang, 421001 China; 2 Department of Anesthesiology, West China Hospital, Sichuan University, Chengdu, China

**Keywords:** Lactylation, Kla, Breast cancer, Prognosis, Immunotherapy

## Abstract

**Background:**

Histone lysine lactylation (*Kla*) is a newly identified histone modification, which plays a crucial role in cancer progression. Hence, we determined the prognostic value of Kla in breast cancer (BC).

**Methods:**

We obtained RNA expression profiles of BC from The Cancer Genome Atlas (TCGA), following screening out Kla-specific genes. Furthermore, we determined the prognostic value of Kla by constructing a cox model based on Kla-specific genes. Subsequently, we identified expression of lactate accumulation-related genes and prognostic Kla-specific genes through Human Protein Atlas (*HPA*), and further performed a correlation analysis based on their expression. Meanwhile, we explored the effects of Kla on BC tumor microenvironment (TME), drug therapy and immunotherapy. Moreover, we predicted the pathways influenced by Kla via gene set enrichment analysis (GSEA).

**Results:**

A total of 1073 BC samples and 112 normal controls were obtained from TCGA, and 23 tumor samples were removed owing to inadequate clinical information. We identified 257 differentially expressed Kla-specific genes (*DEKlaG*s) in BC. A cox model involved with *CCR7*, *IGFBP6*, *NDUFAF6*, *OVOL1* and *SDC1* was established, and risk score could be visualized as an independent biomarker for BC. Meanwhile, *Kla* was remarkably associated with BC immune microenvironment, drug therapy and immunotherapy. *Kla* was identified to be related to activation of various BC-related KEGG pathways.

**Conclusion:**

In conclusion, *Kla* contributes to drug resistance and undesirable immune responses, and plays a crucial role in BC prognosis, suggesting that *Kla* was expected to be a new therapeutic target for BC.

**Supplementary Information:**

The online version contains supplementary material available at 10.1186/s12920-023-01726-1.

## Introduction

Breast cancer (BC) is a heterogeneous disease with high level of mortality, and it is the fifth leading reason of cancer-associated death [1]. BC has surpassed lung cancer as the most prevalent malignancy in 2020 [2], and it is characterized by local recurrence, distant metastasis and chemotherapy resistance, which are the major causes that lead to the high mortality of BC patients [3]. Although advances in BC prevention, diagnosis and personalized therapy in accordance with molecular classification [4, 5], therapeutic targets for BC are still lacking, which contributes to unfavorable prognosis. Therefore, it is crucial to determine more effective therapeutic targets for improving the overall survival of BC patients.

Histone posttranslational modifications have been identified to play a vital role in cancer progression, antitumor immunity and therapy [6–8]. Feng et al. indicated that histone posttranslational modifications can contribute to maintaining genome stability, transcription, DNA repair, and chromatin modulation in BC [9]. Recently, Zhao et al. identified a new histone posttranslational modification type, called histone lysine lactylation (*Kla*) [10], which could stimulate or inhibit gene transcription from chromatin directly. Lactate is predominantly derived from aerobic glycolysis, a characteristic of cancer cells [11], and always accumulates in the tumor microenvironment (TME). Studies showed that lactate could promote cancer local invasion, metastasis [12], and inhibit immune response [13]. Lactate in TME promoted the development of myeloid-derived suppressor cells (MDSCs) [13] and modulated dendritic cell activation, which might remarkably contribute to tumor escape [14]. Moreover, lactate derived from tumor could inhibit tumor surveillance by T and NK cells, which led to tumor immune escape [15]. Recently, the roles of *Kla* on malignancies have attracted more attention since identified by Zhao et al [16]. Majority of researches showed that aberrant *Kla* level was associated with tumorigenesis and malignant progression [17, 18]. In addition, inhibition of histone *Kla* could impair the tumorigenicity of cancer stem cells [19]. BC is characterized by activation of aerobic glycolysis [20, 21], leading to accumulation of lactate in the TME. However, there is no study to evaluate the carcinogenic role of Kla in BC.

In our study, we downloaded gene expression profiles from The Cancer Genome Atlas (TCGA), following screening out differentially expressed *Kla*-specific genes (*DEKlaG*s). Subsequently, *DEKlaG*s were enrolled in univariate and multivariate cox regression analyses to build a risk model. Furthermore, we evaluated the prognostic value of *Kla*-specific genes, and then determined the contribution of *Kla* to BC TME, drug therapy and immunotherapy. Finally, gene set enrichment analysis (GSEA) revealed the potential mechanisms of *Kla* in BC.

## Materials and methods

### Data preparation

In 2019, Zhao et al. identified the newly posttranslational modification histone Kla, and then determined the Kla-specific genes via ChIP-seq. Hence, we downloaded all of the Kla-specific genes from Zhao’s study [10]. BC *RNA* expression profiles and their corresponding clinical data were downloaded from TCGA (https://portal.gdc.cancer.gov/), including 1073 BC samples and 112 normal controls. All of the IHC image data were obtained from Human Proteins Atlas (*HPA*) database (Table S1) (https://www.proteinatlas.org/).

### To identify prognostic value on *Kla*

The expression levels of *Kla*-specific genes were extracted, following differentially expressed analysis in R software limma package, with the cut-off criteria |log_2_(Fold-Change)| >=1 and p-value < 0.05. To identify the prognostic value of *Kla*, univariate cox analysis was used to screen prognostic genes, following constructing cox model via multivariate cox analysis according to prognostic *Kla*-specific genes. According to cox model, BC patients were divided into high- and low-risk groups on basis of risk score median. And we further validated the accuracy of cox formula via survival analysis and independent prognostic analysis. In addition, the prognostic value of genes enrolled in cox model was also determined.

### Correlation analysis between lactate accumulation related genes and *Kla* specific genes

According to previous study, E1A binding protein p300 (P300) was regarded as a writer of *Kla*. In addition, Zhao et al. indicated that lactate dehydrogenase A (*LDHA*), lactate dehydrogenase B (*LDHB*) and hypoxia inducible factor 1 subunit alpha (*HIF1A*) also played a crucial role in *Kla* process. Therefore, we evaluated the expression of these four genes, and the correlation analyses between these four genes and prognostic *Kla*-specific genes were determined in BC.

### Tumor microenvironment (TME) analysis

Firstly, immune cells’ levels of BC patients were calculated via “CIBERSORT” in R software. The correlations between prognostic *Kla*-specific genes and immune cells were performed. In addition, immune scores of BC patients in TCGA were gained through single sample gene set enrichment analysis (ssGSEA) in packages “GVSA” and “GSEAbase” of R software. Then, we further explored the relationship of immune cell scores, immune function and *Kla*-specific genes. Subsequently, we downloaded the stemness score data according to DNA methylation (DNAss) and RNA (RNAss) from UCSC Xena database (http://xena.ucsc.edu/). Stemness score correlation analysis was further determined.

### Tumor mutation burden (TMB) correlation analysis

Tumor mutation burden (TMB), the number of mutations which exist in a tumor and are related to the emergence of neoantigens that trigger antitumor immunity, is identified as a new indicator for prediction of response to immunotherapy [22]. Hence, we downloaded the TMB data from UCSC Xena (https://xena.ucsc.edu/), and then explored the relevance between TMB and *Kla*-specific genes in BC.

### Immunotherapy and immune checkpoint analysis

To further explore the relationship between *Kla* and immunotherapy, we obtained the immunotherapy data from the TCIA database (https://tcia.at/home). Subsequently, we analyzed the correlation between prognostic *Kla*-specific genes and immunotherapy in BC. In addition, we acquired the immune checkpoint data from previous publications [23], and then we explored the relevance of *Kla* and checkpoints.

### Drug susceptibility analysis

Drug susceptibility data were downloaded from the CellMiner database (https://discover.nci.nih.gov/cellminer/home.do). Furthermore, the effects of *Kla* on BC drug therapy were evaluated via correlation analysis.

### Gene set enrichment analysis (GSEA)

To evaluate the potential mechanism of *Kla* in BC, we explored the potential KEGG pathways influenced by *Kla*-specific genes via Gene Set Enrichment Analysis (GSEA), and the top 3 pathways of each prognostic *Kla*-specific gene were listed.

### Statistical analysis

The software SPSS (Version 23.3, IBM) was used to perform statistical analyses. Pearson’s Correlation Tests, Student’s T-test and long-rank p test were carried out in this study. Significance difference was considered at p < 0.0001****; p < 0.001***; p < 0.01 **; p < 0.05 *.

## Results

### Identification of prognostic value

**Fig. 1:**
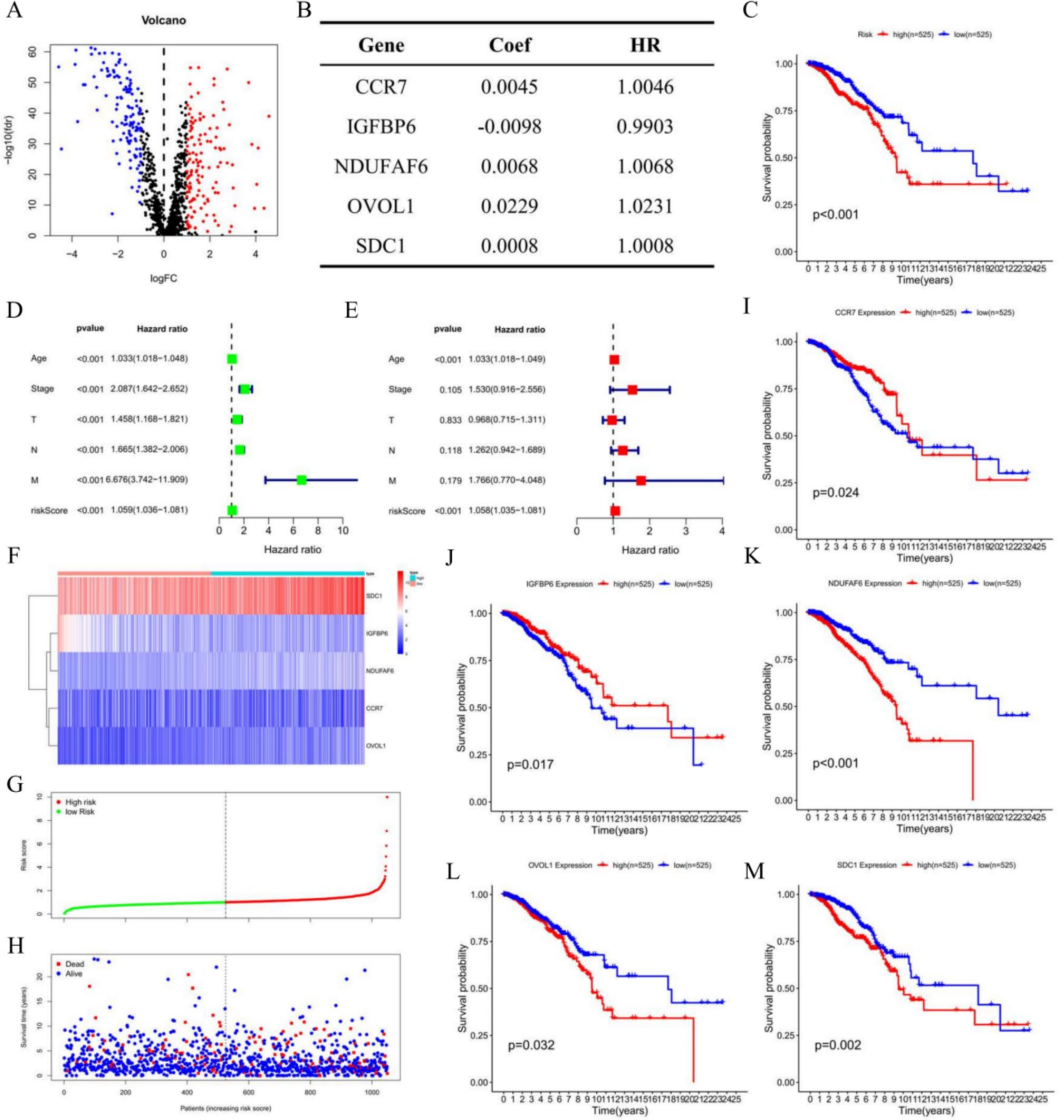
Identification prognostic value of *Kla*-specific genes. **A**, Differentially expressed *Kla*-specific genes (*DEKlaG*s) in BC, with the cut-off criteria |log2FC|>=1, p-value < 0.05. **B**, Cox regression model in accordance with *DEKlaG*s. **C**, Survival analysis according to risk score calculated by *Kla*-specific genes expression. **D**;**E**, Independent prognostic analysis of risk score. T represents the tumor size in tumor TNM classification, N represents the lymph node metastasis in TNM classification, and M represents distant metastasis in TNM classification. **F**, Visualization of risk level combined with gene expression. Heatmap represents the prognostic gene expression profiles. Blue stands for low expression, while red stands for high expression. The type means risk level. **G**, Distribution of each risk score according to Kla-specific genes. Green represents low-risk group, while red represents high-risk group. **H**, Visualization of survival time and risk score. Patients with high-risk score tend to have shorter survival time. **I-M**, Survival analysis of prognostic *Kla*-specific genes. FC, Foldchange

According to differentially expressed analysis, we screened out 257 differentially expressed Kla-specific genes (*DEKlaG*s) with the cut-off criteria |log_2_FC| >=1 and p-value < 0.05 (Fig. 1A, Table S2) in BC. To explore the prognostic value of *DEKlaG*s, we selected prognostic *Kla*-specific genes via univariate cox analysis (Table 1), and then built a cox model through multivariate cox regression analysis (Fig. 1B). Furthermore, risk score of each patient was calculated based on C-C Chemokine Receptor 7 (*CCR7*), insulin like growth factor binding protein 6 (*IGFBP6*), NADH: ubiquinone oxidoreductase complex assembly factor 6 (*NDUFAF6*), ovo like transcriptional repressor 1 (*OVOL1*) and syndecan 1 (*SDC1*) expression level, following dividing into low- and high-risk groups on basis of risk median, respectively. Survival analysis showed that high-risk patients had unsatisfied overall survival compared to low-risk group (Fig. 1C, Figure S1). In addition, prognostic value analysis indicated that risk score in accordance with *Kla* might be an independent prognostic biomarker for BC (Fig. 1D, E). Risk score combined with gene expression profiles, survival time were visualized in R (Fig. 1F, G, H). Furthermore, the prognostic value of *Kla*-specific genes enrolled in cox formula was also identified (Fig. 1I-M).

**Table 1:** Prognostic Kla-specific genes in BRCA

Gene	HR	HR.95 L	HR.95 H	coxPvalue
CCR7	1.0038	1.0008	1.0068	0.0133
IGFBP6	0.9900	0.9812	0.9989	0.0272
IL27	1.2274	1.0094	1.4926	0.0400
NDUFAF6	1.0095	1.0021	1.0169	0.0119
OVOL1	1.0284	1.0022	1.0554	0.0336
SDC1	1.0008	1.0003	1.0012	0.0007

### Identification of *Kla*-specific genes expression

**Fig. 2:**
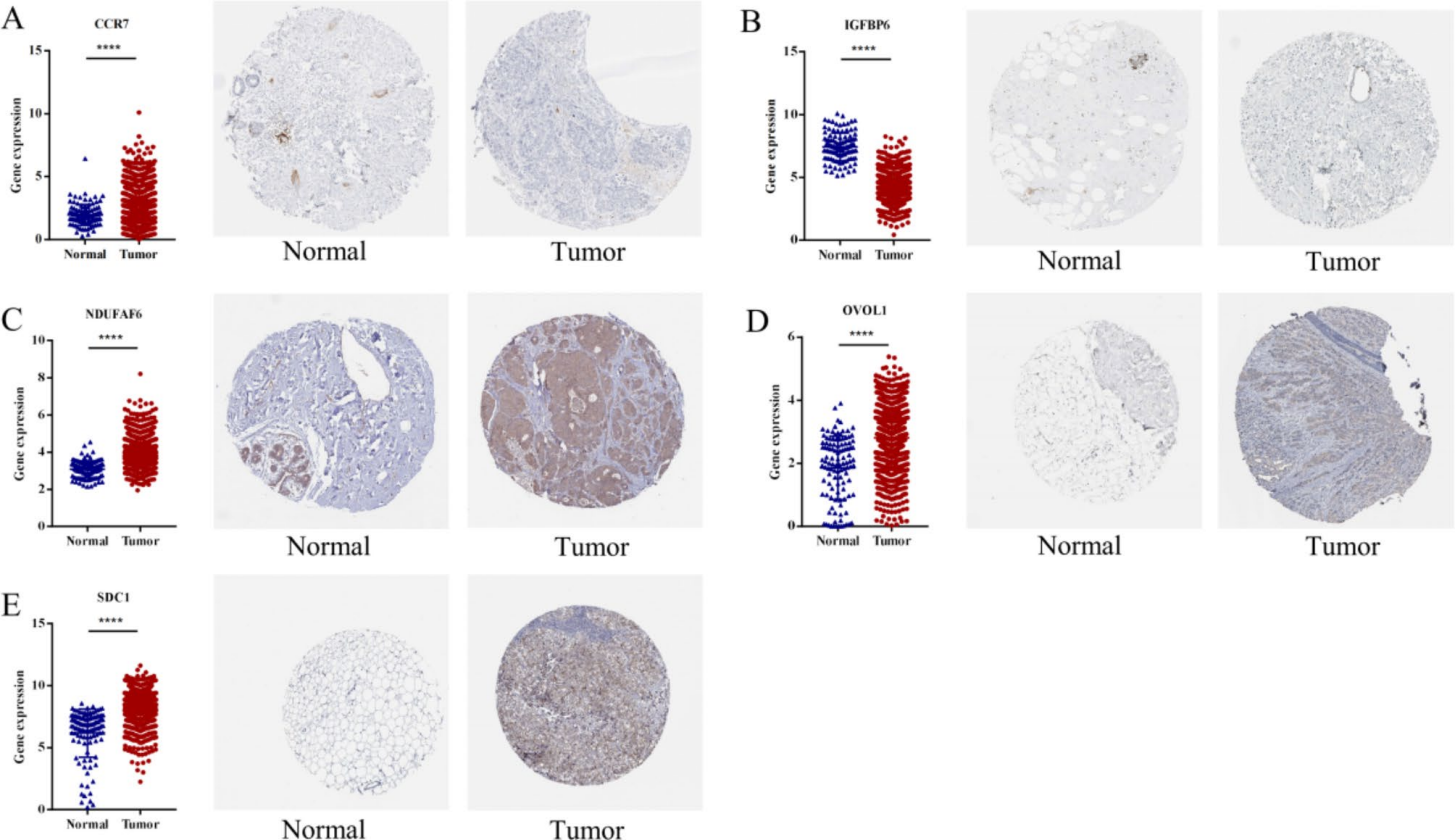
Identification of *Kla*-specific genes expression. *RNA* and protein expression of *CCR7* (**A**), *IGFBP6* (**B**), *NDUFAF6* (**C**), *OVOL1* (**D**) and *SDC1* (**E**) in BC were obtained from TCGA and HPA database, respectively

According to *Kla*-specific genes enrolled in cox model, we further evaluated their *RNA* and protein expression in BC. As the results shown, *CCR7 RNA* expression level in TCGA was overexpressed in tumor samples. However, the protein expression based on IHC in HPA was opposite (Fig. 2A). And patients with high *CCR7* expression had favorable overall survival (Fig. 1G). The potential mechanism should be further explored. The RNA levels of *IGFBP6*, a tumor suppressor gene in BC, were downregulated in BC samples. Meanwhile, the protein expression of IGFBP6 was nearly not detected in tumor tissues (Fig. 2B). *NDUFAF6*, *OVOL1* and *SDC1*, as oncogenes, were all upregulated in BC samples (Fig. 2C, D, E).

### Identification of lactate-related genes in BC

**Fig. 3:**
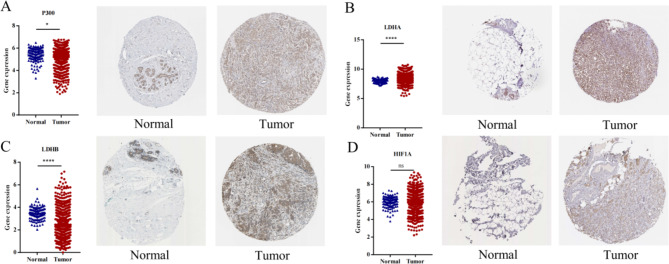
Identification of lactate accumulation related genes in BC. The expression of *P300* (**A**), *LDHA* (**B**), *LDHB* (**C**) and *HIF1A* (**D**) in BC cases

Zhao et al. indicated that the 4 genes *P300, LDHA, LDHB and HIF1A* were related to lactate accumulation and *Kla* modification [10]. Therefore, we explored the expression of these 4 genes in BC. The results showed that *P300, LDHA and LDHB* were all overexpressed in tumor samples (Fig. 3A, B, C). Although *RNA* level had no significance between normal controls and BC patients, *HIF1A* protein was significantly upregulated in BC (Fig. 3D).

### Correlation analysis between lactate accumulation-related genes and *Kla* -specific genes

**Fig. 4:**
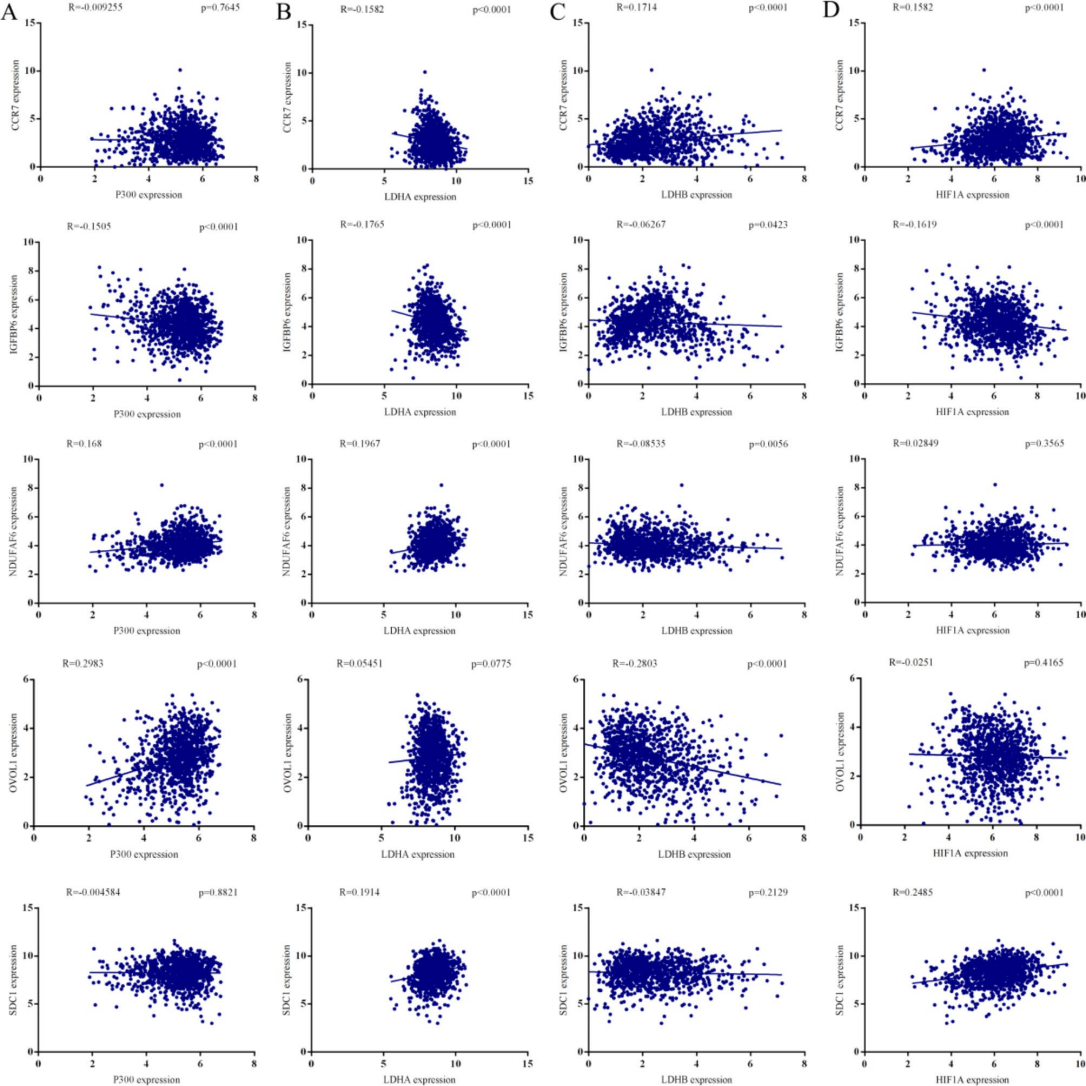
Correlation between *Kla*-specific genes and lactate accumulation related genes. Correlation between *P300* (**A**), *LDHA* (**B**), *LDHB* (**C**), *HIF1A* (**D**) and *Kla*-specific genes including *CCR7, IGFBP6, NDUFAF6, OVOL1* and *SDC1*.

Lactate accumulation-related genes were all identified to overexpression in BC. We further explored the relevance between lactate accumulation-related genes and prognostic *Kla*-specific genes. As the figure shown, *P300* was positively related to *NDUFAF6* and *OVOL1*, and negatively related to tumor suppress gene *IGFBP6* (Fig. 4A). *HIF1A* was associated with *CCR7*, *IGFBP6* and *SDC1* (Fig. 4B). *LDHA*, overexpression in BC, was positively relevant to oncogenes *NDUFAF6, OVOL1* and *SDC1*, while negatively relevant to *CCR7* and *IGFBP6* (Fig. 4C). *LDHB* only played a promoted role in *CCR7* expression, and played an inhibited role in other 4 genes (Fig. 4D). Taken together, the expression of tumor suppressor gene IGFBP6 in BC was negatively associated with Kla production, suggesting that *IGFBP6* might be a crucial Kla target for BC.

### *Kla* was associated with immunity in BC TME

**Fig. 5:**
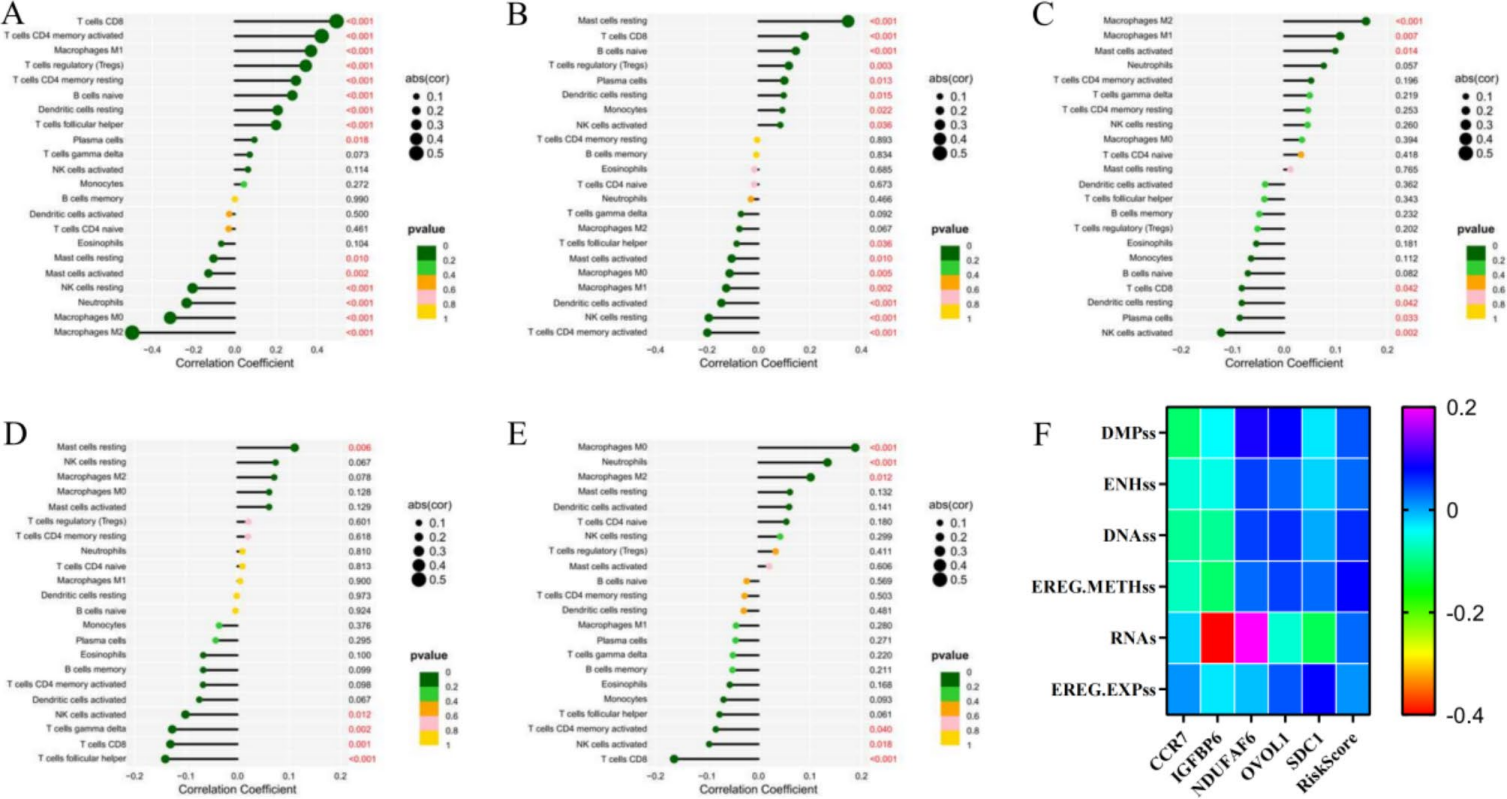
Correlation between *Kla*-specific genes and immune microenvironment. *CCR7* (**A**), *IGFBP6* (**B**), *NDUFAF6* (**C**), *OVOL1* (**D**) and *SDC1* (**E**) expression were correlated with immune cells level in BC. (**F**), Heatmap of correlation between *Kla* and stemness. DNAss: DNA methylation-based, EREG-METHss: Epigenetically regulated DNA methylation-based, DMPss: Differentially methylated probes-based, ENHss: Enhancer Elements/DNAmethylation-based; RNAss: RNA expression-based, EREG.EXPss: Epigenetically regulated RNA expression-based

To evaluate the role of *Kla* on immunity, we determined the relevance between *Kla*-specific genes and various immune cells. *CCR7* expression was significantly related to majority of immune cells level. The most positively and negatively relevant immune cell type were T cell CD8 and Macrophage M2 which was identified to contribute to cancer progression, respectively (Fig. 5A). And *IGFBP6* expression was most positively related to Mast cells resting, and negatively related to T cells CD4 memory activated (Fig. 5B). *NDUFAF6*, *OVOL1* and *SDC1*, as oncogenes in BC, were all positively related to Macrophage M2, while negatively related to NK cells activated (Fig. 5C, D, E). Furthermore, according to the immune cell scores and immune function from *ssGSEA*, we determined the difference between high and low expression group of there 5 genes (Figure S2). In addition, *NDUFAF6, OVOL1* and *SDC1* were positively related, while *CCR7* and *IGFBP6* were negatively related to stemness score in BC (Fig. 5F).

### *Kla* was related to BC TMB

**Fig. 6:**
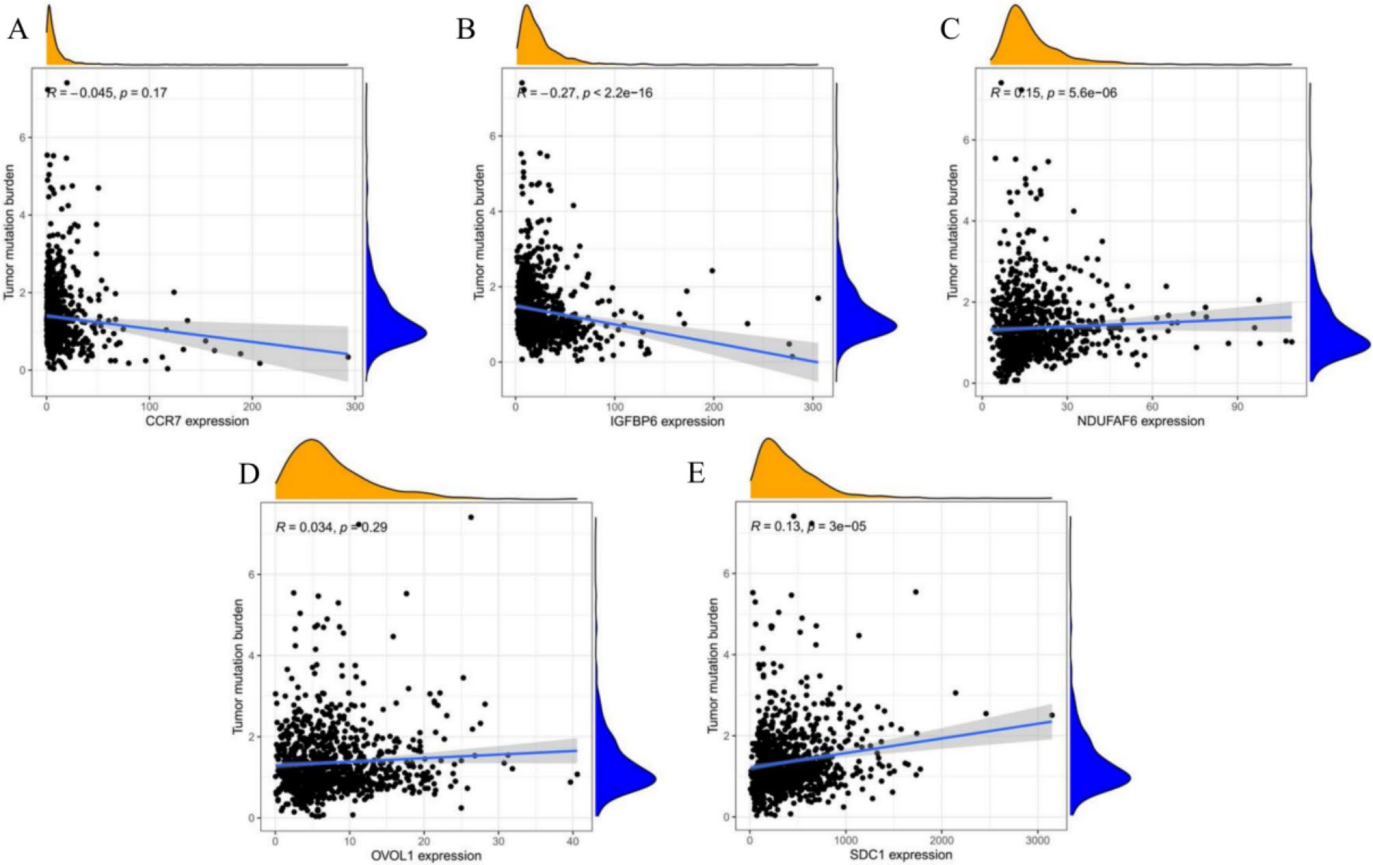
Correlation between *Kla* and *TMB*. High *CCR7* (**A**) and *IGFBP6* (**B**) level meant lower TMB in BC. High *NDUFAF6* (**C**), *OVOL1* (**D**) and *SDC1* (**E**) meant high TMB level

TMB was regarded as a new indicator for the response to immunotherapy. Therefore, we explored the relationship between TMB and *Kla*. As the results shown, *CCR7* had no effect on TMB in BC (Fig. 6A), while high *IGFBP6* expression always meant low level of TMB (Fig. 6B). Oncogenes *NDUFAF6, OVOL1* and *SDC1* were all positively related to TMB level in BC (Fig. 6C, D, E), indicating that *Kla* might play a crucial role in BC immunotherapy.

### *Kla* was related to BC immunotherapy

**Fig. 7:**
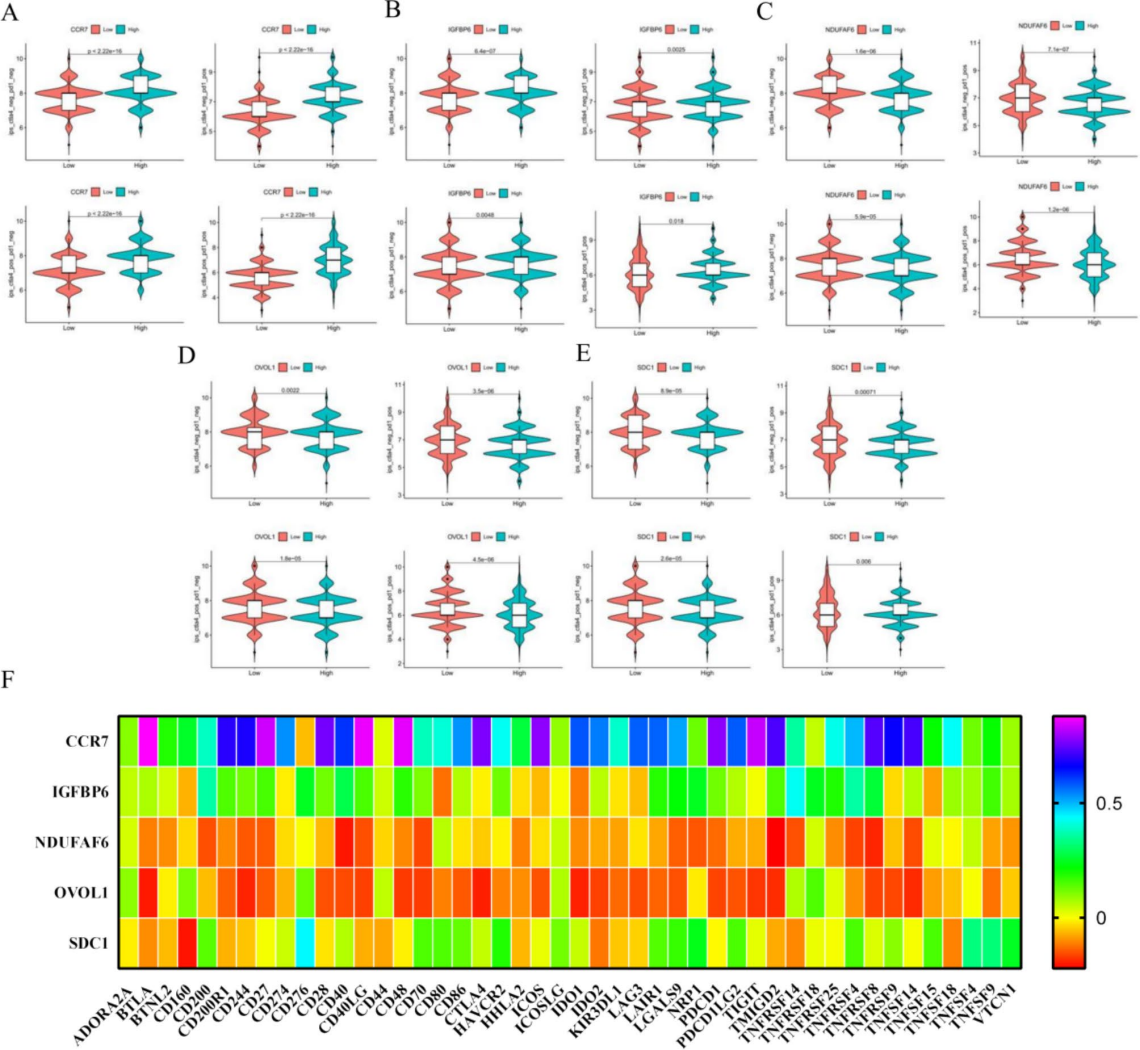
Immunotherapy analysis on basis of *Kla*. **A-E**, *CCR7* and *IGFBP6* were positively related to immunotherapy response, while *NDUFAF6, OVOL1* and *SDC1* were opposite. F, the correlation between *Kla* and immune checkpoint expression

To further determine the role of *Kla* on BC immunotherapy, we downloaded the immunotherapy information of BC samples from TCIA. The results showed that BC patients with high *CCR7* and *IGFBP6* expression had more favorable immunotherapy response than low expression (Fig. 7A, B). Conversely, as oncogenes, *NDUFAF6*, *OVOL1* and *SDC1* played an inhibited role in immunotherapy process (Fig. 7C, D, E). Furthermore, we explored the correlation between *Kla*-specific genes and immune checkpoints. The results showed that *CCR7* and *IGFBP6* were positively relevant to nearly all checkpoints, while *NDUFAF6, OVOL1* and *SDC1* were opposite (Fig. 7F).

### Drug susceptibility analysis

**Fig. 8:**
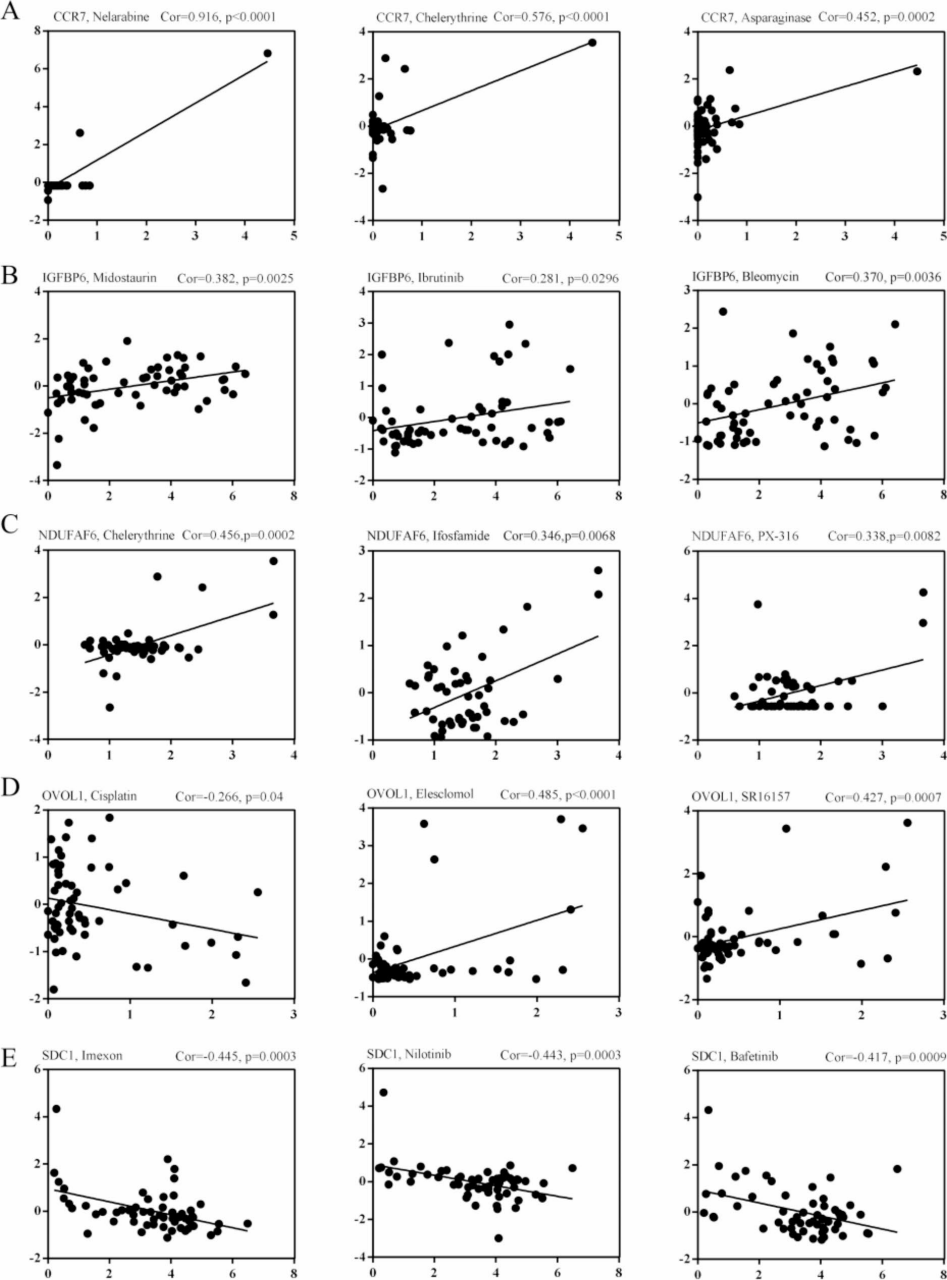
Drug susceptibility analysis. The role of *CCR7* (**A**), *IGFBP6* (**B**), *NDUFAF6* (**C**), *OVOL1* (**D**) and *SDC1* (**E**) on BC drug resistance

To determine the effects of *Kla* on BC drug therapy, we obtained drug susceptibility data, and then analyzed the correlation between drug susceptibility and prognostic *Kla*-specific genes (Tables 2, 3, 4, 5 and 6). High *CCR7* presented high susceptibility in majority of drugs, such as Nelarabine and Chelerythrine (Fig. 8A). Similarly, tumor suppressor gene *IGFBP6* was also associated with the response of drug therapy (Fig. 8B). *NDUFAF6*, as an oncogene, was positively related to drug susceptibility (Fig. 8C). The potential mechanism was unclear. *OVOL1* and *SDC1* displayed a remarkably inhibited role in BC drug therapy, such as Carboplatin, Cisplatin, Nilotinib, Imexon, etc. (Fig. 8D, E).

**Table 2:** Drug susceptibility analysis according to CCR7

Drug	cor	pvalue	Drug	cor	pvalue
Nelarabine	0.9164	< 0.0001	Cytarabine	0.3359	0.0087
Fluphenazine	0.6381	< 0.0001	Melphalan	0.3317	0.0096
Dexamethasone Decadron	0.6148	< 0.0001	Decitabine	0.3291	0.0102
Chelerythrine	0.5764	< 0.0001	Thiotepa	0.3289	0.0103
PX-316	0.5061	< 0.0001	Digoxin	0.3234	0.0117
Asparaginase	0.4518	0.0003	Triethylenemelamine	0.3189	0.0130
Fludarabine	0.4292	0.0006	XK-469	0.3164	0.0138
Hydroxyurea	0.4201	0.0008	Etoposide	0.3140	0.0146
Cyclophosphamide	0.4063	0.0013	Seliciclib	-0.3042	0.0181
Pipobroman	0.3800	0.0027	Cladribine	0.2983	0.0206
Fenretinide	0.3724	0.0034	Calusterone	0.2963	0.0215
Chlorambucil	0.3641	0.0042	LMP-400	0.2791	0.0308
Dexrazoxane	0.3573	0.0051	Nitrogen mustard	0.2770	0.0321
Ifosfamide	0.3486	0.0063	Carmustine	0.2724	0.0353
Arsenic trioxide	0.3481	0.0064	LY-294,002	-0.2651	0.0407
Idarubicin	0.3423	0.0074	Teniposide	0.2647	0.0409
Batracylin	0.3401	0.0079	Raltitrexed	0.2594	0.0453
Uracil mustard	0.3365	0.0086			

**Table 3:** Drug susceptibility analysis according to IGFBP6

Drug	cor	pvalue	Drug	cor	pvalue
Midostaurin	0.3823	0.0026	Tyrothricin	-0.2775	0.0318
Bleomycin	0.3698	0.0036	Dolastatin 10	-0.2817	0.0292
Staurosporine	0.3524	0.0058	Cyclophosphamide	-0.2867	0.0264
Dasatinib	0.3448	0.0070	Hypothemycin	-0.3047	0.0179
Floxuridine	0.3374	0.0084	Lapachone	-0.3080	0.0167
Simvastatin	0.3141	0.0145	Actinomycin D	-0.3160	0.0139
Irofulven	0.2921	0.0236	Tamoxifen	-0.3222	0.0121
Ibrutinib	0.2810	0.0296	Eribulin mesilate	-0.3315	0.0097
5-fluoro deoxy uridine 10mer	0.2740	0.0341	Vinblastine	-0.3343	0.0090
Itraconazole	0.2572	0.0472	Nilotinib	-0.3514	0.0059
Imatinib	-0.2548	0.0494	Vinorelbine	-0.3553	0.0053
Cobimetinib (isomer 1)	-0.2570	0.0474	Bafetinib	-0.3693	0.0037
Raloxifene	-0.2578	0.0468	Pipamperone	-0.3736	0.0033
Selumetinib	-0.2605	0.0444	Arsenic trioxide	-0.3763	0.0030
Paclitaxel	-0.2664	0.0396			

**Table 4:** Drug susceptibility analysis according to NDUFAF6

Drug	cor	pvalue
Nelarabine	0.4567	0.0002
Chelerythrine	0.4561	0.0002
Vorinostat	0.3993	0.0016
Ifosfamide	0.3457	0.0068
PX-316	0.3379	0.0083
Belinostat	0.3048	0.0179
Amonafide	0.2746	0.0338

**Table 5:** Drug susceptibility analysis according to OVOL1

Drug	cor	pvalue
Elesclomol	0.4850	0.0001
SR16157	0.4267	0.0007
bisacodyl, active ingredient of viraplex	0.4052	0.0013
Fluorouracil	0.3687	0.0037
Fulvestrant	0.3632	0.0043
By-Product of CUDC-305	0.3595	0.0048
Acetalax	0.3547	0.0054
Carboplatin	-0.3364	0.0086
Simvastatin	-0.3099	0.0160
Staurosporine	-0.2887	0.0253
kahalide f	0.2852	0.0272
Arsenic trioxide	-0.2836	0.0281
Bleomycin	-0.2795	0.0306
Tegafur	0.2792	0.0307
Raloxifene	0.2723	0.0353
Benzimate	0.2702	0.0368
Midostaurin	-0.2675	0.0388
Pyrazoloacridine	0.2671	0.0391
Cisplatin	-0.2656	0.0403
Cordycepin	0.2624	0.0428
Testolactone	-0.2562	0.0481
Carmustine	-0.2552	0.0491

**Table 6:** Drug susceptibility analysis according to SDC1

Drug	cor	pvalue	Drug	cor	pvalue
Imexon	-0.4446	0.0004	Dacarbazine	-0.2953	0.0220
Nilotinib	-0.4434	0.0004	Pipamperone	-0.2896	0.0248
Chelerythrine	-0.4260	0.0007	Ixazomib citrate	-0.2896	0.0248
Arsenic trioxide	-0.4180	0.0009	Selumetinib	-0.2820	0.0290
Bafetinib	-0.4174	0.0009	XK-469	-0.2782	0.0314
Cyclophosphamide	-0.4149	0.0010	ABT-199	-0.2765	0.0325
Hypothemycin	-0.4078	0.0012	Bendamustine	-0.2728	0.0349
Lapachone	-0.3699	0.0036	Imatinib	-0.2618	0.0433
Dimethylaminoparthenolide	-0.3690	0.0037	BN-2629	-0.2584	0.0462
Carmustine	-0.3545	0.0055	Oxaliplatin	-0.2571	0.0474
Nelarabine	-0.3522	0.0058	Irofulven	0.2683	0.0382
Vorinostat	-0.3501	0.0061	Dasatinib	0.2746	0.0337
Ifosfamide	-0.3475	0.0065	Itraconazole	0.2772	0.0320
Bortezomib	-0.3459	0.0068	Everolimus	0.2965	0.0214
Lomustine	-0.3113	0.0155	kahalide f	0.3064	0.0173

### Enrichment pathway of prognostic kla-specific genes

**Fig. 9:**
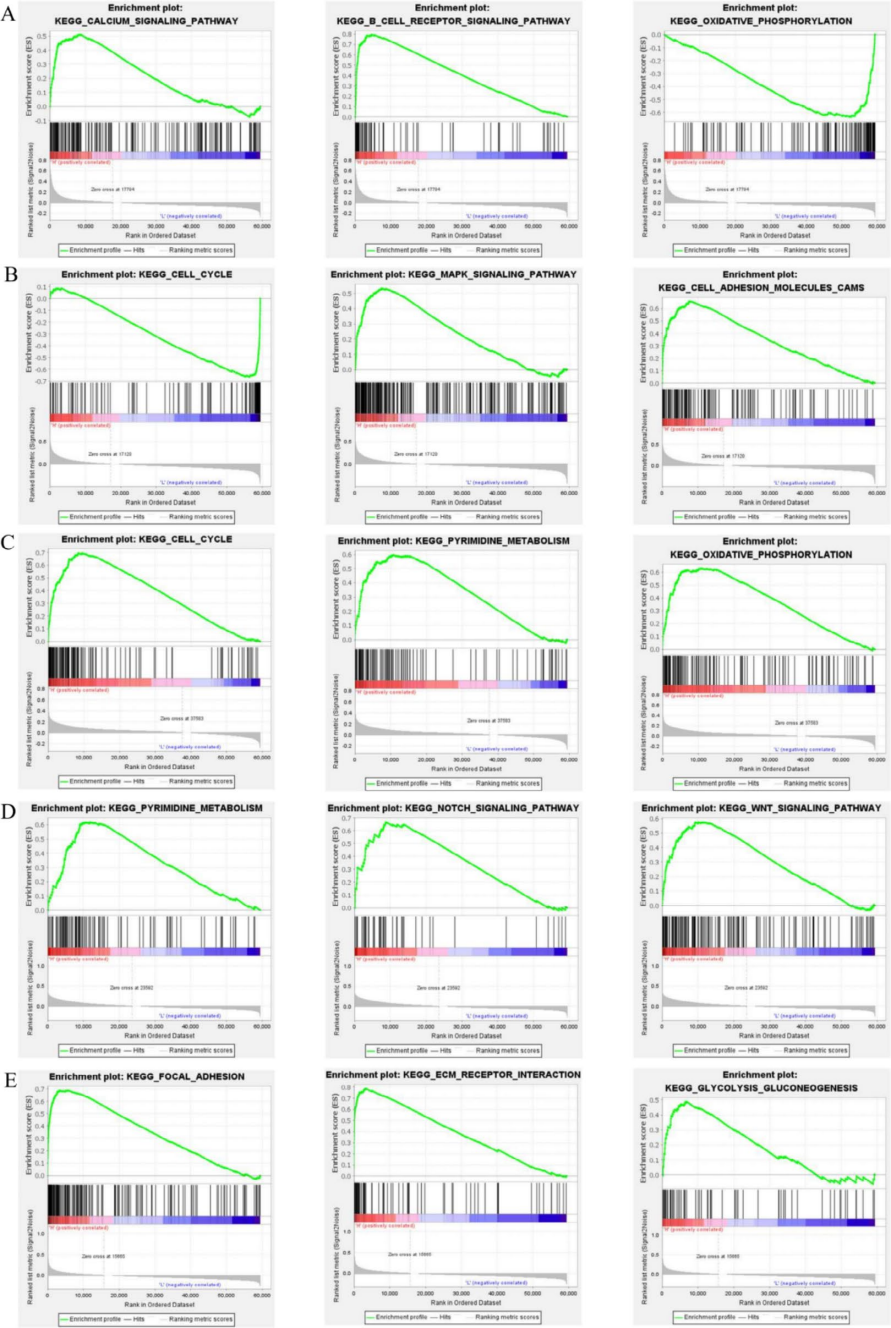
Gene set enrichment analysis. *KEGG* pathways influenced by *CCR7* (**A**), *IGFBP6* (**B**), *NDUFAF6* (**C**), *OVOL1* (**D**) and *SDC1* (**E**) in BC. The horizontal axis represents the sequenced genes, while the vertical axis represents the corresponding running enrichment score (ES). The peak is the ES of this gene set. The black vertical lines are the target genes in the gene set. The genes before the peak were the core genes in the gene set, indicating the genes that contributed the most to the final ES of the pathway. The red meant bigger logFC, while blue is opposite

To explore the potential KEGG pathways influenced by *Kla*, we carried out *GSEA*, and showed that *CCR7* was related to immune response pathways, such as B cell receptor signaling pathway. And it also played a negative role in BC cancer cell oxidative phosphorylation process (Fig. 9A). *IGFBP6* inhibited the activity of cell cycle and alanine aspartate and glutamate metabolism pathways. But as a tumor suppressor gene, *IGFBP6* was associated with activation of *MAPK* signaling pathway (Fig. 9B). *NDUFAF6* played a crucial role in the activation of cell cycle and oxidative phosphorylation (Fig. 9C). *OVOL1* and *SDC1* were also related to activation of several cancer related pathways, such as *NOTCH*, *WNT* signaling pathways and focal adhesion (Fig. 9D, E).

## Discussion

Normal cells always produce energy via mitochondrial oxidative phosphorylation, while cancer cells, owing to massive energy demands, are characterized by reprogramming metabolic pathways such as aerobic glycolysis [12]. Activation of aerobic glycolysis plays a crucial role in BC tumorigenesis and progression [24, 25]. Chen et al. indicated that aerobic glycolysis was associated with drug resistance of BC [26]. Generally, aerobic glycolysis leads to accumulation of lactate in the TME, which is related to histone *Kla* and plays a vital role in cancer progression and tumor immunity [27, 28]. However, whether lactate produced by aerobic glycolysis and histone *Kla* play a carcinogenic role in BC is unclear. Therefore, we determined the role of *Kla* in BC.

In present study, we built a cox model to predict BC patient prognosis, and the risk score in accordance with prognostic *Kla*-specific genes could be regarded as an independent prognostic biomarker. 2 tumor suppressor genes including *CCR7, IGFBP6* and 3 oncogenes including *NDUFAF6, OVOL1, SDC1* were involved in cox model. *CCR7* was one of chemokine receptors identified be upregulated in BC. Signals mediated by *CCR7* can activate T and B lymphocytes, and regulate the migration of immune cells to inflamed tissue [29]. In 2001, A Müller et al. demonstrated that *CCR7* was upregulated in BC and played a vital role in determining the metastatic destination of tumor cell [30]. In addition, in a BC mouse model, downregulation of *CCR7* might impair the tumor cell proliferation and invasive properties, indicating that *CCR7* might promote distant metastasis via promoting tumor cell proliferation and invasion at the metastatic site [31]. Philippe A Cassier et al. demonstrated that *CCR7* was expressed by spindle shaped stromal cells in BC, but its expression showed no difference on patient overall survival [32]. Taken together, although studies suggest that *CCR7* seems to reliably predict the lymph node metastases of BC, it is unclear whether *CCR7* can be associated with BC patient survival. In our study, *RNA* expression of *CCR7* was elevated in TCGA BC samples. However, patients with high *CCR7* expression had favorable prognosis. High *CCR7* expression always meant high immune cell and immune function scores. Patients with high *CCR7* level had better responses to drug therapy and immunotherapy. The potential mechanism is unclear. More studies, of course, should be carried out to explore the function of *CCR7* in BC. In the future, it will be important to correlate the types of cells that express *CCR7* in BC with stage of progression. *IGFBP6* was associated with cell migration and positive regulation of stress-activated *MAPK* cascade [33]. *IGFBP6* was regarded as a biomarker of BC [34]. Knockdown of *IGFBP6* was more resistant to apoptosis and increased the proliferation of cancer cells. Meanwhile, BC with low *IGFBP6* expression had a high probability of metastasis due to a more efficient invasion of tumor cells [35]. In our study, we identified *IGFBP6* as a tumor suppressor gene, which played a positive role in BC drug therapy and immunotherapy. BC patients with high *IGFBP6* expression always meant lower risk level and high overall survival rate. It was also found that upregulation of IGFBP6 was positively related to high immune cell scores, such as NK cells and TILs. Elevation of IGFBP6 also promoted the immune process, especially Type-II-IFN response, and responses to immunotherapy, suggesting that IGFBP6 might be a candidate immunotherapeutic target for BC. We identified that Kla production was negatively related to *IGFBP6* expression, but Lucia Longhitano et al. indicated that lactate could enhance the expression of *IGFBP6*, and then induce the microglia M2 polarization in glioblastoma [36], and *IGFBP6* induced by lactate promoted glioblastoma cells migration and colony formation. Meanwhile, stimulation with lactate in BC cells led to upregulation of *IGFBP6*, which was controversial with our study. *IGFBP6* could also induce expression of various genes related to mitochondrial biogenesis, and then promote cancer cell proliferation [37], which was controversial with previous studies [35, 38]. Moreover, Shkurnikov MY showed that *IGFBP6* could correctly predict the emergence of BC relapse with sensitivity of more than 80%, and poor prognosis was related to low expression *IGFBP6* [39, 40]. In conclusion, the role of *IGFBP6* in BC was controversial, and more studies should be performed to evaluate its biological function and effect on drug therapy and immunotherapy. *NDUFAF6* is relevant to assembly of complex I (NADH-ubiquinone oxidoreductase) in the mitochondrial respiratory chain via regulation of subunit *ND1* biogenesis [41]. Recently, Lu HJ et al. indicated that *NDUFAF6* was identified as a potential prognostic gene in hepatocellular carcinoma (HCC) via bioinformatics analysis, and showed promise to be a new therapeutic target. In BC, Lu et al. suggested that *NDUFAF6*, as a lactate metabolism gene, was most related to BC prognosis, and played a crucial role in NK cells activation [42], which was similar to our study. We also suggested that *NDUFAF6* contributed to cell cycle and oxidative phosphorylation in BC. *NDUFAF6* might inhibit the function of various immune cells and immune responses. Meanwhile, overexpression of *NDUFAF6* was associated with high TMB level and undesirable immunotherapy response. *NDUFAF6* was also negatively related to various immune checkpoint expression in BC, indicating that it showed promise to be an immunotherapy target for BC. *OVOL1* was identified to overexpression in BC, and related to activation of several BC-related pathways, such as *NOTCH* and *WNT* signaling pathways [43, 44]. However, Drug susceptibility analysis showed that it correlated with drug response, such as Elesclomol and SR16157. Fan CN et al. identified that *OVOL1* could impair *TGF-β/SMAD* signaling and maintain the epithelial identity of BC cells [45]. Therefore, *OVOL1* might act as a tumor suppressor gene in BC, and it is necessary to carry out more studies to further explore its effect on BC immunotherapy. *SDC1*, an integral membrane protein, participates in cell proliferation, cell migration and cell-matrix interactions through its receptor for extracellular matrix proteins [46]. Yang et al. suggested that targeting *SDC1* might be a new opportunity for cancer therapy [46]. In pancreatic ductal adenocarcinoma (PDAC), serum *SDC1* level was remarkably elevated, and receiver operating characteristic (ROC) analysis area under the curve was 0.847 [47], suggesting that serum *SDC1* served as a promising novel biomarker for PDAC early diagnosis. It was found that *SDC1* was associated with malignant tumor metastasis and drug resistance [48]. In our study, we identified that *SDC1* contributed to focal adhesion of BC, and negatively correlated with immune responses, especially Type-II-IFN response. Meanwhile, high *SDC1* level meant high Macrophage M2 and low NK cell activation, which all played a crucial role in BC metastasis and immunotherapy [49–51]. Our further TMB correlation analysis, drug susceptibility and immunotherapy analysis validated the results, which were similar to previous studies [52]. In addition, Juliana Maria Motta et al. indicated that *SDC1* showed promise to be a candidate target for therapeutic strategies against BC [53]. However, fewer studies focused on *SDC1* to explore its mechanism and effect on BC immunotherapy. In conclusion, these *Kla*-specific genes were associated with the initiation and progression of BC, and also played a crucial role in BC TME, drug therapy and immune process, indicating that histone *Kla* might be a potential therapeutic target for BC.

## Conclusion

In present study, we investigated the prognostic value of *Kla* in BC by cox regression analysis, and showed that Kla might be a potential independent prognostic biomarker for BC. It was also found that *Kla* production was associated unfavorable prognosis of BC patients, and played a crucial role in BC TME, drug resistance and immunotherapy responses. Finally, we suggested Kla production might induce the activation of various BC-accociated KEGG pathways. These findings showed that *Kla* was expected to be a new therapeutic target for BC.

### Electronic supplementary material

Below is the link to the electronic supplementary material.

Supplementary Material 1

Supplementary Material 2

Supplementary Material 3

Supplementary Material 4

## Data Availability

The datasets analyzed during the current study are available from the corresponding author on reasonable request.

## Abbreviations

Kla: Lysine lactylation
BC: Breast cancer
TCGA: The Cancer Genome Atlas
HPA: Human Protein Atlas
TME: Tumor microenvironment
GSEA: Gene set enrichment analysis
DEKlaGs: Differentially expressed Kla-specific genes
MDSCs: Myeloid-derived suppressor cells
LDHA: Lactate dehydrogenase A
LDHB: Lactate dehydrogenase B
HIF1A: Hypoxia inducible factor 1 subunit alpha
SsGSEA: Single sample gene set enrichment analysis
TMB: Tumor mutation burden
DEGs: Differentially expressed genes
CCR7: C- Chemokine Receptor 7
IGFBP6: Insulin like growth factor binding protein 6
NDUFAF6: Ubiquinone oxidoreductase complex assembly factor 6
OVOL1: Ovo like transcriptional repressor 1
SDC1: Syndecan 1
HCC: Hepatocellular carcinoma
PDAC: Pancreatic ductal adenocarcinoma
ROC: Receiver operating characteristic
KEGG: Kyoto Encyclopedia of Genes and Genomes
