# Supplementary material for: Lysine lactylation (Kla) might be a novel therapeutic target for breast cancer
Source: BMC Med Genomics. 2023 Nov 10;16:283. doi: 10.1186/s12920-023-01726-1 (PMC10636881; doi:10.1186/s12920-023-01726-1)
Supplement: Supplementary file 1 — Supplementary Material 1 [file 12920_2023_1726_MOESM1_ESM.doc]

**Fig.S1**
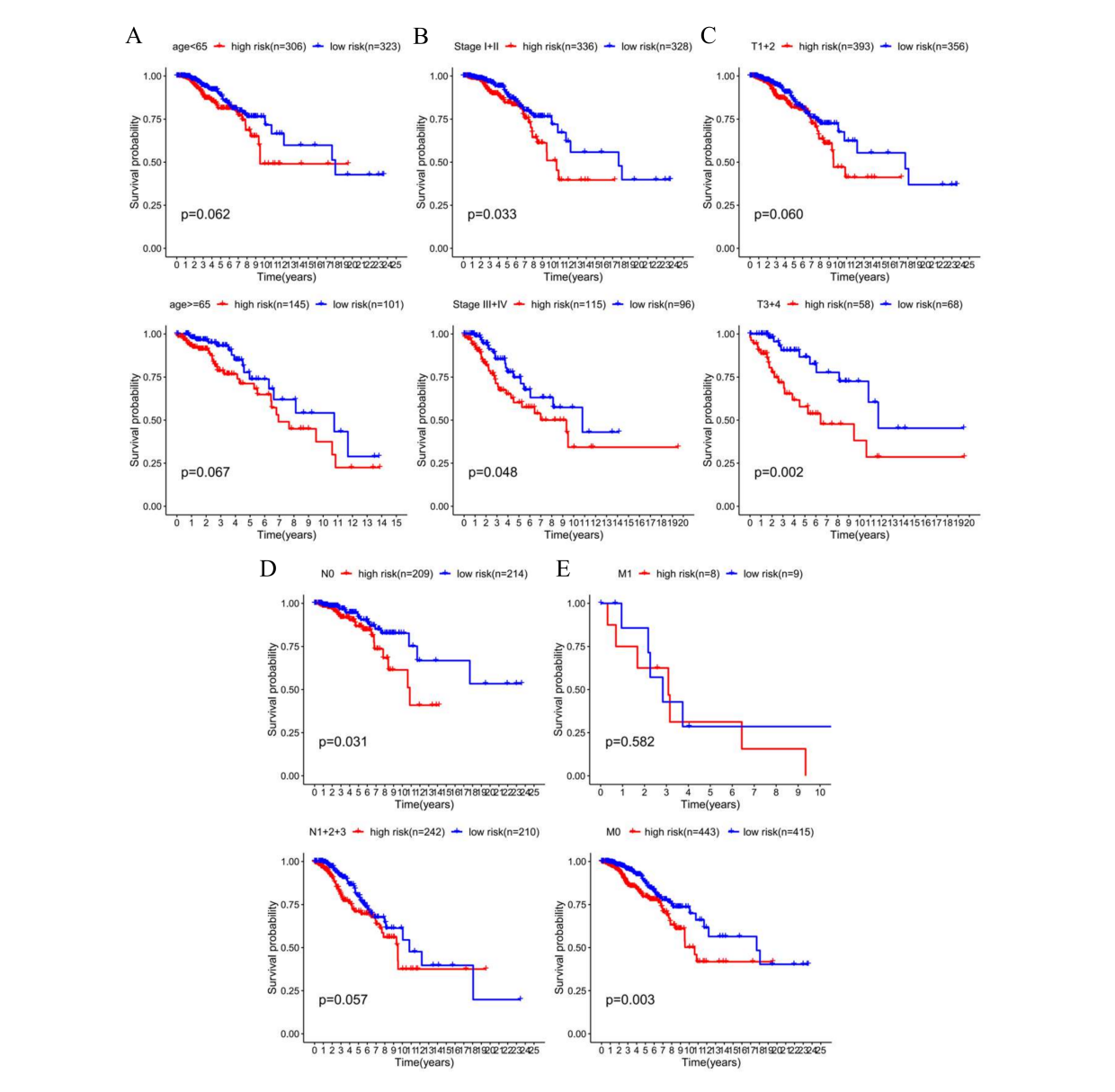
**Fig.S1** Survival analysis combined with stratification analysis of clinical parameters including Age (A), Stage (B), T (C), N (D) and M (E) classification in TNM system.
