# Supplementary material for: Lysine lactylation (Kla) might be a novel therapeutic target for breast cancer
Source: BMC Med Genomics. 2023 Nov 10;16:283. doi: 10.1186/s12920-023-01726-1 (PMC10636881; doi:10.1186/s12920-023-01726-1)
Supplement: Supplementary file 2 — Supplementary Material 2 [file 12920_2023_1726_MOESM2_ESM.doc]

**Fig.S2**


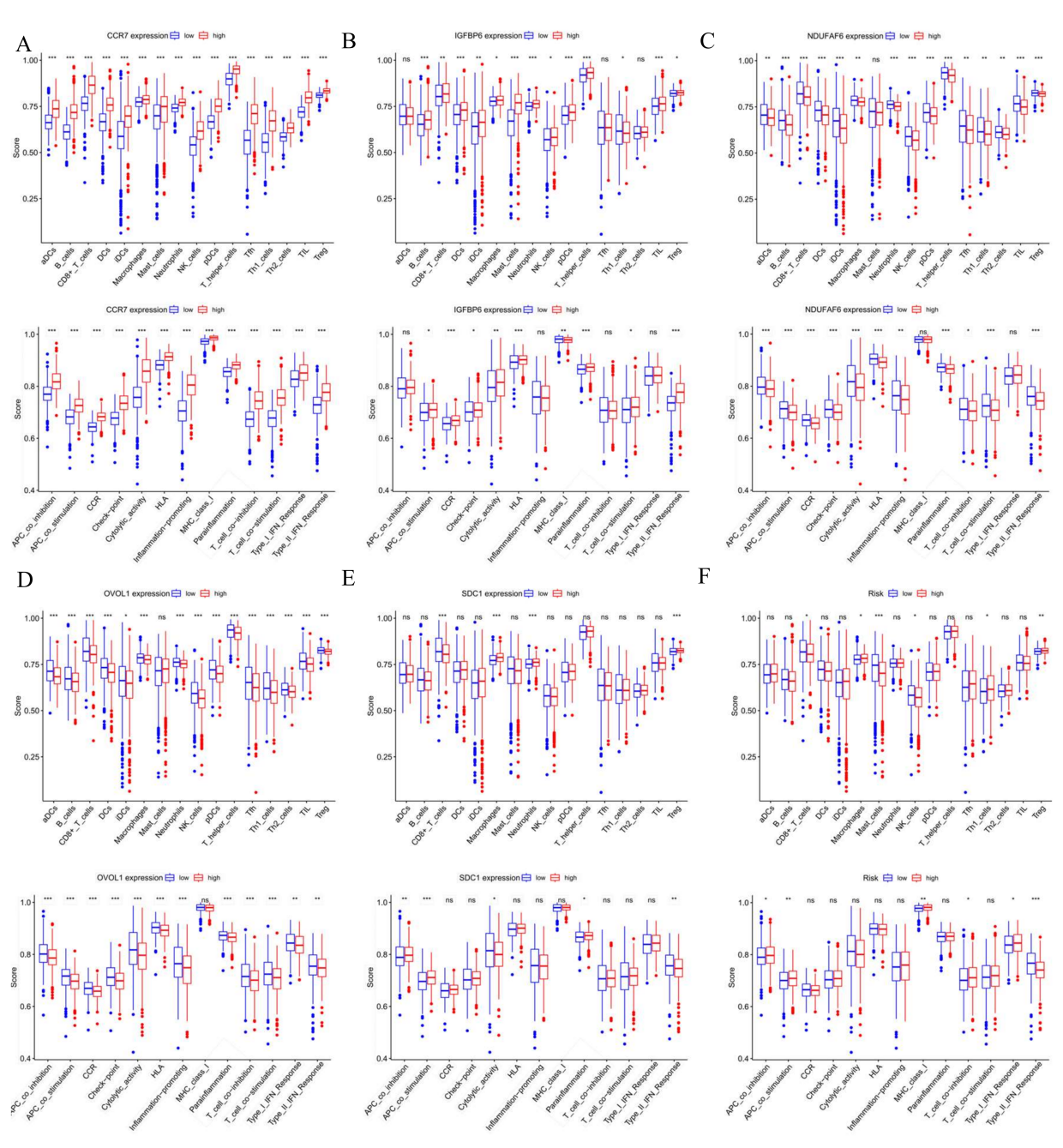
**Fig.S2** The effect of *Kla*-specific genes on BC TME. A-F, The prognostic *Kla*-specific genes expression including *CCR7* (A), *IGFBP6* (B), *NDUFAF6* (C), *OVOL1*(D), *SDC1* (E) and risk score (F) were correlated with immune cells and immune cell function scores.
