# Supplementary material for: Lysine lactylation (Kla) might be a novel therapeutic target for breast cancer
Source: BMC Med Genomics. 2023 Nov 10;16:283. doi: 10.1186/s12920-023-01726-1 (PMC10636881; doi:10.1186/s12920-023-01726-1)
Supplement: Supplementary file 3 — Supplementary Material 3 [file 12920_2023_1726_MOESM3_ESM.doc]

| **Table S1 Antibody information in HPA database** | |
| --- | --- |
| **Protein-Name** | **Antibody information** |
| CCR7 | CAB003796 |
| IGFBP6 | HPA008005 |
| NDUFAF6 | HPA047148 |
| OVOL1 | HPA003984 |
| SDC1 | CAB002424 |
| P300 | HPA004112 |
| LDHA | CAB069404 |
| LDHB | CAB004641 |
| HIF1A | HPA001275 |
