# Supplementary material for: Lysine lactylation (Kla) might be a novel therapeutic target for breast cancer
Source: BMC Med Genomics. 2023 Nov 10;16:283. doi: 10.1186/s12920-023-01726-1 (PMC10636881; doi:10.1186/s12920-023-01726-1)
Supplement: Supplementary file 4 — Supplementary Material 4 [file 12920_2023_1726_MOESM4_ESM.doc]

| **Table S2 Differentially expressed Kla-specific genes in BRCA** | | | | | | | |
| --- | --- | --- | --- | --- | --- | --- | --- |
| **Gene** | **logFC** | **Gene** | **logFC** | **Gene** | **logFC** | **Gene** | **logFC** |
| ABCA5 | -1.4893 | EGLN3 | 1.5248 | MAMDC2 | -2.9908 | RNASE10 | 2.2760 |
| ABCA6 | -2.4710 | EHD2 | -1.7417 | MAML2 | -1.8879 | RNASEH2A | 1.6681 |
| ABHD2 | 1.0178 | EIF3L | -1.0810 | MARCO | -1.4526 | RND1 | 1.5025 |
| ACSS2 | -1.4857 | ENO2 | 1.4646 | ME1 | -1.3867 | RNF157 | -1.5059 |
| ACTA2 | -1.7092 | EPHA2 | -1.1919 | MEIS3 | 1.0008 | ROGDI | 1.5736 |
| ADAM33 | -2.6249 | F10 | -2.4512 | METRN | 2.3167 | RTN4R | 1.2065 |
| ADAMTSL4 | -1.5556 | FABP4 | -4.5806 | MFSD4A | -1.0505 | RUNDC3A | 2.8712 |
| ADGB | 1.9027 | FADS6 | 2.9384 | MICAL3 | -1.0234 | RUNDC3B | -1.6117 |
| ADHFE1 | -1.4991 | FAM149A | -1.9528 | MID1 | -1.4389 | RUSC1 | 1.5143 |
| ADM | -1.4342 | FAM222A | 1.1079 | MMP10 | 2.9852 | S100A11 | 1.1501 |
| ADORA2A | 1.0627 | FAM43A | -1.1031 | MMP28 | -1.8315 | SBK2 | 4.3605 |
| AFAP1L1 | -1.3477 | FBXO40 | -4.4608 | MMP8 | 1.3597 | SCN3B | -2.1976 |
| AHRR | 1.0570 | FCRLB | 3.0941 | MMP9 | 2.6172 | SCRIB | 1.0072 |
| AIFM2 | -1.5700 | FHOD3 | -1.3708 | MRGBP | 1.0322 | SDC1 | 1.7974 |
| AIM2 | 1.7804 | FILIP1L | -1.1964 | MRPL24 | 1.0958 | SELP | -1.6206 |
| ALDOC | -1.8476 | FLRT2 | -1.6109 | MS4A4A | -1.0470 | SERPINF1 | -1.0777 |
| APLP1 | 2.3667 | FLYWCH2 | 1.1793 | MTFR2 | 2.7567 | SEZ6L2 | 1.3330 |
| APOO | 1.1377 | FNDC10 | 1.5881 | MYOM1 | -3.8283 | SGK1 | -1.4555 |
| ARID5B | -1.4390 | FXYD2 | -2.0240 | NAT14 | 1.5812 | SIGIRR | 1.1313 |
| ARMC3 | 2.7574 | GALR3 | 1.1449 | NAT8L | -2.9029 | SLC11A1 | 1.4133 |
| ASB4 | -1.8814 | GEM | -1.0070 | NDUFAF6 | 1.2293 | SLC13A2 | -1.9467 |
| ATF3 | -2.0437 | GINS4 | 1.8970 | NFE2 | 1.3707 | SLC16A7 | -3.0441 |
| B4GALT3 | 1.2000 | GIPC2 | -2.5763 | NKAIN1 | 4.1077 | SLC22A18 | 1.1891 |
| BCL9 | 1.2679 | GNG7 | -1.2733 | NMB | -1.7493 | SLC25A22 | 1.4939 |
| BIK | 1.4825 | GPR141 | 1.9674 | NXNL1 | -2.9241 | SLC37A1 | 1.1706 |
| BST1 | -1.0365 | GPR18 | 1.3169 | OIT3 | 1.6881 | SLC6A9 | 1.4897 |
| C1QTNF6 | 2.1809 | GPR68 | 1.7270 | OLFML2B | 1.2817 | SLC7A5 | 2.4748 |
| CABLES2 | 1.2118 | GPRC5B | -1.2041 | OLR1 | 2.1616 | SLC7A8 | 1.1292 |
| CACNA1B | 2.3702 | GPX3 | -3.4313 | OVOL1 | 1.1352 | SOX5 | -1.4733 |
| CACNA1D | 1.3380 | GRK3 | -1.5046 | P2RY14 | -1.6101 | SPAG1 | 1.3274 |
| CACNB3 | 1.3668 | GRTP1 | 1.0459 | PALM | -1.1578 | SPEF1 | 2.3560 |
| CARD6 | -1.1764 | GSTM2 | -1.2185 | PDE1B | -1.5045 | SSX2IP | 1.1942 |
| CASP12 | -2.1516 | HAS1 | -2.2493 | PDE7B | -1.5032 | ST18 | 1.2285 |
| CAV1 | -3.1720 | HBEGF | -1.0172 | PER2 | -1.2371 | STARD10 | 2.0407 |
| CBX7 | -1.4621 | HCN2 | 3.6914 | PHLDB1 | -1.1484 | STBD1 | -1.4466 |
| CCDC189 | 1.4679 | HDAC11 | 1.0509 | PI16 | -3.5058 | SUSD2 | 1.0795 |
| CCL24 | -2.2434 | HOGA1 | -1.6216 | PID1 | -2.3479 | SYN1 | 1.9004 |
| CCND2 | -1.0219 | HSD11B1 | -2.2071 | PIWIL4 | -1.1719 | SYNPO | -1.6599 |
| CCR7 | 1.6208 | HSD17B7 | 1.2211 | PKIB | 1.7183 | TAL2 | 1.0778 |
| CD34 | -1.9602 | HSPB1 | 1.5886 | PKP3 | 1.1202 | TBC1D30 | 1.0971 |
| CD36 | -3.8669 | ICAM2 | -1.3842 | PLPP7 | -1.4454 | TBC1D31 | 1.2289 |
| CDKN1C | -1.9564 | IFITM1 | 1.1677 | PLPPR3 | 1.7171 | TFPI | -1.6560 |
| CELSR3 | 2.4270 | IFNB1 | 4.0456 | PPBP | -2.0523 | TLCD2 | -1.4574 |
| CFAP45 | 2.6111 | IGFBP6 | -3.1516 | PPFIA4 | 1.2061 | TLE6 | 1.5330 |
| CH25H | -1.0787 | IL12B | 2.1405 | PPM1E | 2.2308 | TMEM82 | 3.9501 |
| CLEC7A | 1.2708 | IL17RD | -1.5299 | PRKCA | -1.0709 | TMOD1 | -2.4394 |
| CLMP | -2.1720 | IL18 | 1.1905 | PROZ | 1.9216 | TNS1 | -2.8079 |
| COBLL1 | -1.1245 | IL21R | 2.5633 | PRUNE2 | -1.3155 | TPBGL | 1.1058 |
| COL23A1 | -1.1498 | IL27 | 1.6279 | PRX | -1.1875 | TPM3 | 1.1561 |
| CPEB1 | -1.3804 | ITM2A | -2.2397 | PSMB3 | 1.1435 | TPPP3 | -1.0351 |
| CREB3L1 | 2.2742 | JAM2 | -1.9588 | PTPRM | -1.3433 | TREM1 | 1.2182 |
| CREB5 | -2.0382 | KCNB1 | -2.7103 | PTPRN | 1.2680 | TREML4 | 1.8637 |
| CRIM1 | -1.5965 | KCNJ10 | 2.2457 | PYCR1 | 2.1845 | TRIB3 | 1.5775 |
| CSF3 | -3.7547 | KDM4B | 1.1534 | RAB33A | 1.0178 | TSPAN13 | 1.5940 |
| CSF3R | 1.0380 | KDM5B | 1.0069 | RALGPS2 | 1.1649 | TUBB3 | 2.8505 |
| CX3CL1 | -2.5145 | KIFC2 | 2.0057 | RASA4 | -1.2503 | UNC5A | 2.1798 |
| CXCL3 | -2.0317 | LAPTM4B | 1.2842 | RASSF7 | 1.5367 | USP2 | -1.0679 |
| CXXC5 | 1.0183 | LIMS2 | -1.9153 | RCN3 | 1.0216 | VGF | 4.5740 |
| CYP39A1 | -1.2191 | LIX1 | 3.0169 | RCOR2 | 2.2350 | VLDLR | -1.6639 |
| DEGS2 | 2.4637 | LMO2 | -1.1275 | RDH12 | 1.0467 | VWF | -1.8721 |
| DNMT3B | 1.4164 | LPAR2 | 1.0212 | RECK | -1.5963 | WDR24 | 1.0491 |
| DST | -2.3860 | LRFN4 | 1.4165 | RGCC | -1.4433 | YDJC | 1.2641 |
| EDNRB | -2.0828 | LRP4 | -1.0176 | RHOF | 1.4354 | ZDHHC2 | -1.0015 |
| EFNA2 | 3.8329 | MAFF | -1.5067 | RIMS3 | -1.5049 | ZFP36 | -2.0666 |
| EGFR | -1.6745 |  |  |  |  |  |  |
